# Low Infiltration of CD8+ PD-L1+ T Cells and M2 Macrophages Predicts Improved Clinical Outcomes After Immune Checkpoint Inhibitor Therapy in Non-Small Cell Lung Carcinoma

**DOI:** 10.3389/fonc.2021.658690

**Published:** 2021-06-04

**Authors:** Liuning Li, Guojie Lu, Yang Liu, Longlong Gong, Xue Zheng, Hongbo Zheng, Weiguang Gu, Lin Yang

**Affiliations:** ^1^ Department of Medical Oncology, Guangdong Provincial Hospital of Chinese Medicine, The Second Clinical Collage of Guangzhou University of Chinese Medicine, Guangzhou, China; ^2^ Department of Thoracic Surgery (Respiratory Center Area 1), Guangzhou Panyu Central Hospital, Guangzhou, China; ^3^ Department of Medicine, Genecast Biotechnology Co., Ltd, Wuxi, China; ^4^ Oncology Department, Nanhai People’s Hospital/Second School of Clinical Medical, Southern Medical University, Guangzhou, China; ^5^ Department of Thoracic Surgery, Shenzhen People’s Hospital/2nd Clinical Medical College of Jinan University, Shenzhen, China

**Keywords:** immune checkpoint inhibitor therapy, non-small cell lung carcinoma, T cells, macrophages, biomarker

## Abstract

**Background:**

Many clinical studies have shown that patients with non-small cell lung carcinoma (NSCLC) can benefit from immune checkpoint inhibitor (ICI) therapy; however, PD-L1 and tumor mutation burden (TMB), which are recommended by the NCCN guidelines, are still insufficient in predicting the response to and prognosis of immunotherapy. Given the widespread use of ICIs, it is important to find biomarkers that can predict immunotherapy outcomes in NSCLC patients, and the exploration of additional effective biomarkers for ICI therapy is urgently needed.

**Methods:**

A total of 33 stage II-IV NSCLC patients were included in this study. We analyzed immune markers in biopsy and surgical tissue resected from these patients before treatment with ICIs. We examined the infiltration of immune cells and expression of PD-L1 in immune cells using fluorescent multiplex immunohistochemistry (mIHC) stained with CD8/CD68/CD163/PD-L1 antibodies.

**Results:**

In this cohort, we observed that the levels of CD8+ T cells, CD8+PD-L1+ T cells, and CD68+CD163+ M2 macrophages in the total region were independent prognostic factors for progression-free survival (PFS) in NSCLC patients treated with ICIs (HR=0.04, P=0.013; HR=17.70, P=0.026; and HR=17.88, P=0.011, respectively). High infiltration of CD8+ T cells and low infiltration of CD8+PD-L1+ T cells throughout the region were correlated with prolonged PFS (P=0.016 and P=0.02, respectively). No statistically significant difference was observed for CD68+CD163+ M2 macrophages. The joint parameters CD8+ high/CD8+PD-L1+ low, CD8+ high/CD68+CD163+ low and CD8+PD-L1+ low/CD68+CD163+ low predicted better PFS than other joint parameters (P<0.01, P<0.01, and P<0.001, respectively), and they also demonstrated stronger stratification than single biomarkers. The response rate of patients with high infiltration of CD8+ T cells was significantly higher than that of those with low infiltration (P<0.01), and the joint parameters CD8+/CD8+PD-L1+ and CD8+/CD68+CD163+ also demonstrated stronger stratification than single biomarkers.

**Conclusions:**

This retrospective study identified the predictive value of CD8+PD-L1+ T cells, CD8+ T cells, and CD68+CD163+ M2 macrophages in NSCLC patients who received ICIs. Interestingly, our results indicate that the evaluation of joint parameters has certain significance in guiding ICI treatment in NSCLC patients.

## Introduction

Clinical trials and studies have reported that PD-1/PD-L1 inhibitors can significantly improve the outcomes of advanced non-small cell lung cancer (NSCLC) patients in recent decades (1–6), and PD-1/PD-L1 inhibitors are recommended as the standard first-line therapy for advanced or metastatic NSCLC by the National Comprehensive Cancer Network (NCCN) guidelines (7). However, only approximately 20% of NSCLC patients have prolonged and durable responses to PD-1/PD-L1 inhibitors (2, 8). Therefore, it is important to identify biomarkers that can predict the immunotherapy outcomes of NSCLC patients.

Previous studies have explored the use of PD-L1, tumor mutational burden (TMB) and tumor-associated immune cells (TAICs) to predict the clinical outcome of immunotherapy (3, 9–13), and based on those studies, PD-L1 and TMB have been included in the NCCN guidelines. However, the current work shows that these factors still have limitations in predicting the clinical outcome of immunotherapy. The NCCN guidelines recommend PD-1/PD-L1 inhibitors as the first-line treatment for advanced NSCLC patients with a PD-L1+ cell rate ≥ 1%; nonetheless, some studies have shown that PD-L1 has limitations in predicting the efficacy of immunotherapy. CheckMate227 found that NSCLC patients who received nivolumab plus ipilimumab therapy as first-line therapy had better overall survival (OS) than those who received chemotherapy, and this outcome was independent of PD-L1 expression (10). In addition, the CheckMate 026 results showed that no difference in treatment efficacy was found between the nivolumab and chemotherapy groups in the population with a PD-L1+ cell rate ≥50% (11). The predictive value of TMB was demonstrated in KEYNOTE-01. However, no such predictive value for TMB was found in an exploratory analysis of KEYNOTE-189 and KEYNOTE-407 (14, 15). Therefore, PD-L1 and TMB are still insufficient in predicting the response to and prognosis of immunotherapy, and further study is needed to explore more effective biomarkers for immunotherapy.

It has been reported that the type of immune cells can influence the clinical outcomes of patients with tumors (16, 17). In addition to TMB and PD-L1, multiple immune cell subsets have been assessed to determine their predictive value for immunotherapy outcomes in NSCLC (12, 13, 18). It was reported that the infiltration of CD8+ T cells was associated with ICI efficacy (12, 19–21). Tumor-associated macrophages (TAMs) are important immune cells in the tumor microenvironment (TME), as they mediate tumor progression by regulating TME (22). The M1 and M2 states are two main phenotypes of macrophages, and different types of macrophages predict opposite survival outcomes (23). However, which types of macrophages are associated with the efficacy of immunotherapy in NSCLC remains uncertain. PD-L1 expressed on tumor cells and macrophages is a negative regulator of T cell responses (24). Liu et al. (25) found that high levels of CD68+PD-L1+ immune cells were associated with prolonged OS in NSCLC patients treated with ICIs. However, few studies have reported the relationship between the expression of PD-L1 on T cells and ICI efficacy. It has been reported previously that PD-L1^high^ CD8+ T cells are functional effector cells (26). However, a recent pancreatic cancer study found that PD-L1+ T cells may have negative effects on adaptive antitumor immunity. Since CD8+ T cells play an important role in the immune system in killing cancer cells, studying PD-L1 expression on T cells may help us to understand the prediction of ICI efficacy in NSCLC. However, few articles have paid attention to the effects of the expression of PD-L1 on T cells and macrophage subsets on the efficacy and prognosis of immunotherapy in NSCLC, which require further investigation.

The aim of this retrospective study was to explore the predictive value of multiple immune cell subsets, including CD8+ T cells, CD68+ macrophages, CD68+CD163+ M2 macrophages, CD68+CD163- M1 macrophages, CD8+PD-L1+ T cells, CD68+PD-L1+ macrophages, CD68+CD163+PD-L1+ M2 macrophages, and CD68+CD163-PD-L1+ M1 macrophages, in tumors, the stroma and the total region in the context of NSCLC treatment with immunotherapy by using multiplex immunohistochemical staining (27).

## Methods and Materials

### Patients

We used a retrospective cohort of stage II-IV NSCLC patients from Guangdong Provincial Hospital of Chinese Medicine and Guangzhou Panyu Central Hospital that consisted of 33 patients from May 2016 to April 2019. The detailed clinicopathological characteristics are summarized in **Table 1**. Among the 33 NSCLC patients, 24 patients (72.7%) had lung adenocarcinoma (LUAD) and 9 patients (27.3%) had lung squamous cell carcinoma (LUSC). Among the NSCLC patients, 26 (78.8%) were treated with a PD-1 inhibitor, 3 (9.1%) were treated with a PD-L1 inhibitor, 3 (9.1%) were treated with a PD-1 inhibitor combined with chemotherapy, and 1 (3.0%) received combined PD-1 and CTLA-4 inhibitor therapy. Twenty-two patients (66.7%) had a smoking history. All patients were EGFR/ALK wild-type. Seven patients (21.2%) received first-line immunotherapy, sixteen patients (48.5%) received second-line immunotherapy, and ten patients (30.3%) received ≥ third-line immunotherapy.

**Table 1:** Clinical characteristics.

Category		n(%)		
		Overall(n = 33)	Response(n = 11)	Non-response(n = 22)
Gender	Male	26(78.8%)	9(81.8%)	17(77.3%)
	Female	7(21.2%)	2(18.2%)	5(22.7%)
Age	>=62	16(48.5%)	7(63.6%)	9(40.9%)
	<62	17(51.5%)	4(36.4%)	13(59.1%)
Cancer type	LUAD	24(72.7%)	7(63.6%)	17(77.3%)
	LUSC	9(27.3%)	4(36.4%)	5(22.7%)
Stage	II	1(3.0%)	0(0%)	1(4.5%)
	III	11(33.3%)	6(54.5%)	5(22.7%)
	IV	20(60.6%)	4(36.4%)	16(72.7%)
	NA	1(3.0%)	1(9.1%)	0(0%)
Smoking history	Yes	22(66.7%)	8(72.7%)	14(63.6%)
	No	11(33.3%)	3(27.3%)	8(36.4%)
Treatment	PD-1 inhibitor	26(78.8%)	8(72.7%)	18(81.8%)
	PD-L1 inhibitor	3(9.1%)	1(9.1%)	2(9.1%)
	PD-1 inhibitor + chemotherapy	3(9.1%)	2(18.2%)	1(4.5%)
	PD-1+CTLA-4 inhibitor	1(3.0%)	0(0%)	1(4.5%)
Treatment time	First-line	7(21.2%)	3(27.3%)	4(18.2%)
	Second-line	16(48.5%)	4(36.4%)	12(54.5%)
	≥Third-line	10(30.3%)	4(36.4%)	6(27.3%)

### Fluorescent Multiplex Immunohistochemistry (mIHC) Analysis

Biopsy tissue and postoperative surgical tissue samples collected before ICI treatment were processed into paraffin blocks and then cut into 4-μm-thick FFPE sections. Staining of the 4-μm FFPE slides was performed by using the Opal Seven-color IHC Kit (NEL797B001KT; PerkinElmer, Massachusetts, USA). The immune markers evaluated included CD8 (ZA-0508, clone SP16; Zsbio; 1:100), CD68 (ZM-0060, clone KP1; Zsbio; 1:400), CD163 (ZM0428, clone 10D6, Zsbio; 1:200), and PD-L1 (CST13684, clone E1L3N, CST, 1:100). Markers were identified and quantified by mIHC. Briefly, sections were cut from tumor tissue, deparaffinized, rehydrated, and washed in tap water before epitope retrieval/microwave treatment (MWT). Endogenous peroxidase activity was blocked using Antibody Diluent/Block (72424205; PerkinElmer, Massachusetts, USA). Protein blocking was performed using Antibody Diluent/Block. One antigen required one round of labeling, including primary antibody incubation, secondary antibody incubation, and TSA visualization, followed by labeling with the next antibody. Slides were scanned using PerkinElmer Vectra (Vectra 3.0.5; PerkinElmer, Massachusetts, USA). The percentage of positively stained cells among all nucleated cells was counted.

### Statistical Analysis

Statistical analyses were performed using the R software program (version 3.6.2, https://www.r-project.org/), SPSS (version 22) and GraphPad Prism 8 software. The Kaplan-Meier method was used to analyze the associations between marker expression and progression-free survival (PFS). PFS was defined as the time elapsed between ICI treatment initiation and tumor progression. The statistical significance of differences between survival curves was assessed with the log-rank test. The chi-square test was used to analyze associations between immune marker expression and response. PFS analyses were performed by the Kaplan-Meier estimator and log-rank test. Multivariate/univariate Cox proportional hazard regression models and logistic regression models were utilized to examine the variables that were significant in the univariate analyses and their associations with the outcome. Variables with a P value <0.1 in the univariate analyses were entered into the multivariate analysis. P < 0.05 was considered significant in all the analyses.

## Results

### Infiltration of Tumor-Associated Inflammatory Cells (TAICs) in NSCLC

**Figure 1:**
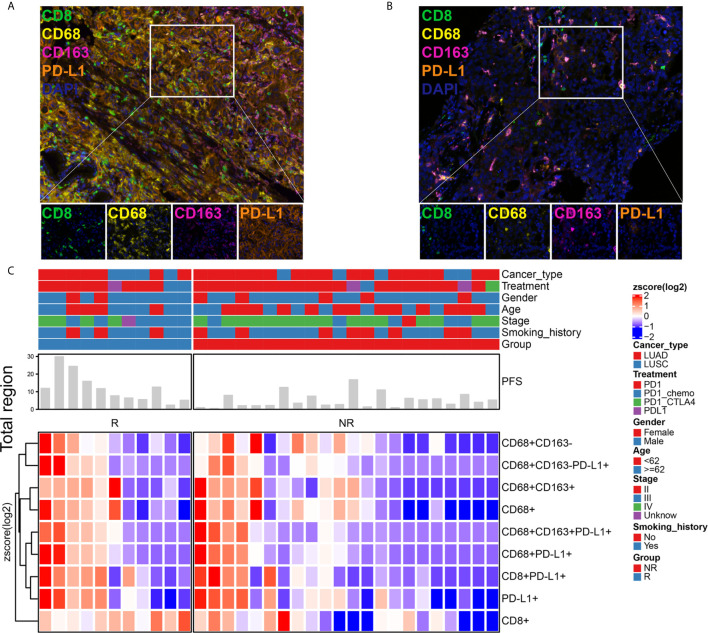
Immune landscape of NSCLC patients treated with immune checkpoint inhibitor (ICI) therapy. Immune cell infiltration was detected using a multiplex immunohistochemistry (mIHC) platform (panel: CD8/CD68/CD163/PD-L1). The mIHC images represent the group that responded to immunotherapy **(A)** and the group that did not respond to immunotherapy **(B)**. Magnification, 200×. The percentages of differentially expressed cells were log-transformed and z-score standardized. Heatmaps of immune cell infiltration in the total region **(C)** were plotted and clustered. They indicated that each patient had a different immune microenvironment, which may lead to different responses to and benefits from immunotherapy. R, response; NR, nonresponse.

We examined a retrospective cohort of 33 patients with stage II-IV NSCLC recruited at Guangdong Provincial Hospital of Chinese Medicine, Nanhai People’s Hospital and Guangzhou Panyu Central Hospital between May 2016 and April 2019, for whom clinical, treatment and extended follow-up data were retrospectively assembled with medical ethics committee approval. Among these patients treated with ICI therapy, we evaluated the infiltration of CD8+ T cells, CD68+ macrophages, CD68+CD163+ M2 macrophages, CD68+CD163- M1 macrophages, CD8+PD-L1+ T cells, CD68+PD-L1+ macrophages, CD68+CD163+PD-L1+ M2 macrophages, and CD68+CD163-PD-L1+ M1 macrophages using mIHC, as shown in **Figures 1A, B**. The immune landscape of NSCLC is shown in **Figures 1C** and **S1**. The percentages of differentially expressed cells were log-transformed and z-score standardized. Heatmaps of immune cell infiltration in the total (**Figure 1C**), stromal (**Figure S1A**) and tumor regions (**Figure S1B**) were plotted and clustered. In the total, stromal and tumor regions, the degree of infiltration of CD8+ T cell, CD68+ TAMs, and PD-L1+ cells were higher in the response [R, response to immunotherapy, including complete response (CR) and partial response (PR)] subgroup than in the nonresponse [NR, nonresponse to immunotherapy, including stable disease (SD) and progressive disease (PD)] subgroup. There were two examples in which a patient who responded to immunotherapy (**Figure 1A**) had more infiltration of immune cells than those who did not respond to immunotherapy (**Figure 1B**). PFS was longer in the R subgroup than in the NR subgroup (**Figure 1C**). This result suggests that the difference in the tumor immune microenvironment may be the reason for the differences in the response rate and PFS of patients who received ICI therapy, and the relationship between TAIC infiltration and PFS needs further study.

### Infiltration of T Cells and M2 Macrophages Were Independent Prognostic Factors

First, to find an “optimal” cutoff value for each marker, the cutoff values for CD8+ T cells, CD68+ macrophages, CD68+CD163+ M2 macrophages, CD68+CD163- M1 macrophages, CD8+PD-L1+ T cells, CD68+PD-L1+ macrophages, CD68+CD163+PD-L1+ M2 macrophages, and CD68+CD163-PD-L1+ M1 macrophages (high vs low) in the total, stromal and tumor regions were determined using the survminer package in R software according to PFS, and the cutoff points are displayed in **Table 2**.

**Table 2:** Cut-off points of all markers according to PFS.

Marker	Cut-off point		
	Total region	Stroma region	Tumor region
CD8+	4.70	6.02	1.35
CD68+	0.48	0.56	0.01
PD-L1+	26.24	17.99	36.19
CD8+PD-L1+	4.57	4.95	3.26
CD68+PD-L1+	3.13	3.16	4.09
CD68+CD163-	0.03	0.06	5.06
CD68+CD163+	6.05	3.82	8.28
CD68+CD163+PD-L1+	1.84	1.76	4.28
CD68+CD163-PD-L1+	1.00	1.57	1.33

**Figure 2:**
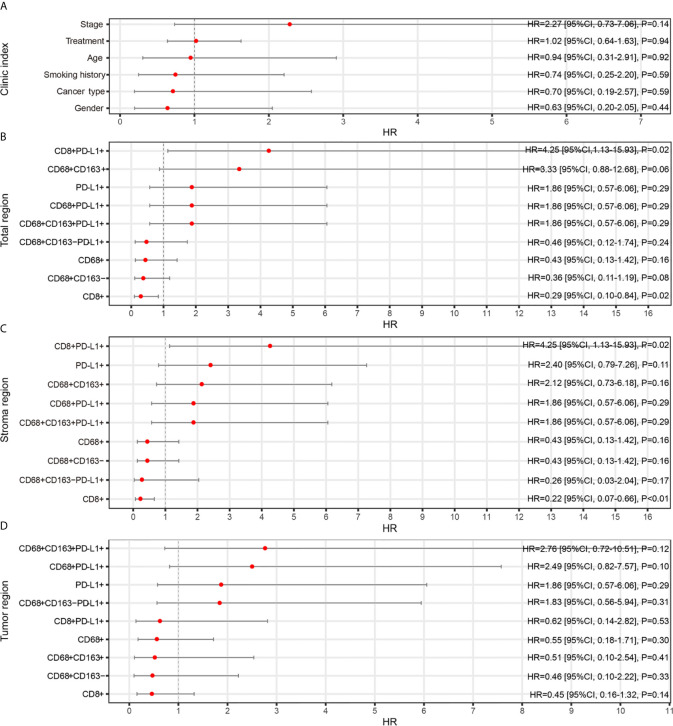
Relationships between TAIC enrichment and progression-free survival (PFS). The forest plot of univariate survival analysis results for clinical indexes **(A)** and enriched TAICs in the total **(B)**, stromal **(C)** and tumor regions **(D)** indicates that high infiltration of CD8+ T cells was a protective factor for prognosis and that high infiltration of CD8+PD-L1+ T cells was a risk factor for prognosis.

**Figure 3:**
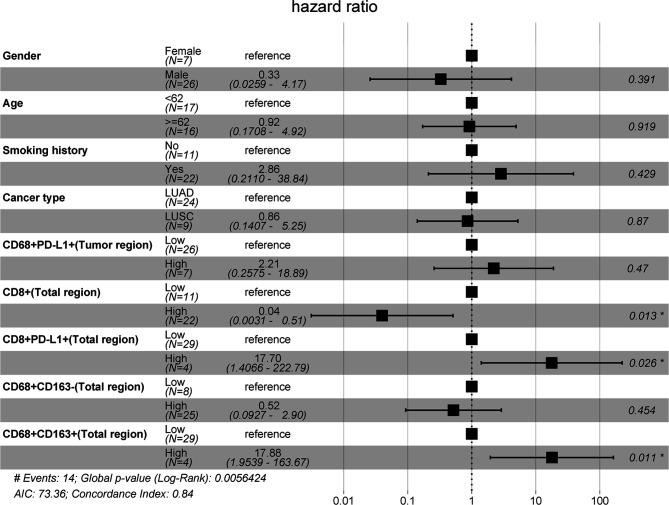
Multivariate Cox model analyses of prognostic factors. The forest plot of multivariate survival analysis results indicates that the levels of infiltrating CD8+, CD8+PD-L1+, and CD68+CD163+ cells in the total region were independent prognostic markers for PFS.

Second, a forest plot of univariate survival analysis results was produced to examine the variables that were significant in the univariate analysis (**Figure 2**). As shown in **Figure 2**, the levels of CD8+ T cells in the total (hazard ratio [HR]=0.29 [95% CI, 0.10-0.84], P =0.02) and stromal regions (HR=0.22 [95% CI, 0.07-0.66], P < 0.01) were correlated with PFS, and the levels of CD8+ PD-L1+ T cells in the total (HR=4.25 [95% CI, 0.13-15.93], P =0.02) and stromal regions (HR=4.25 [95% CI, 0.13-15.93], P =0.02) also revealed significant associations with PFS. In a multivariate Cox analysis, we included clinical parameters and screened the indicators with P ≤ 0.1 in the forest plot of the univariate survival analysis results and a correlation coefficient less than 0.8 (**Figure S2**). The multivariate Cox analysis results showed that CD8+ T cells, CD8+PD-L1+ T cells, and CD68+CD163+ M2 macrophages in the total region were independent prognostic factors for PFS in NSCLC patients treated with ICIs (HR=0.04 (0.0031-0.51), P=0.013; HR=17.70 (1.4066-222.79), P=0.026; and HR=17.88 (1.9539-163.67), P=0.011, respectively; **Figure 3**).

### Prognostic Roles of TAICs and Joint TAIC-Based Parameters

**Figure 4:**
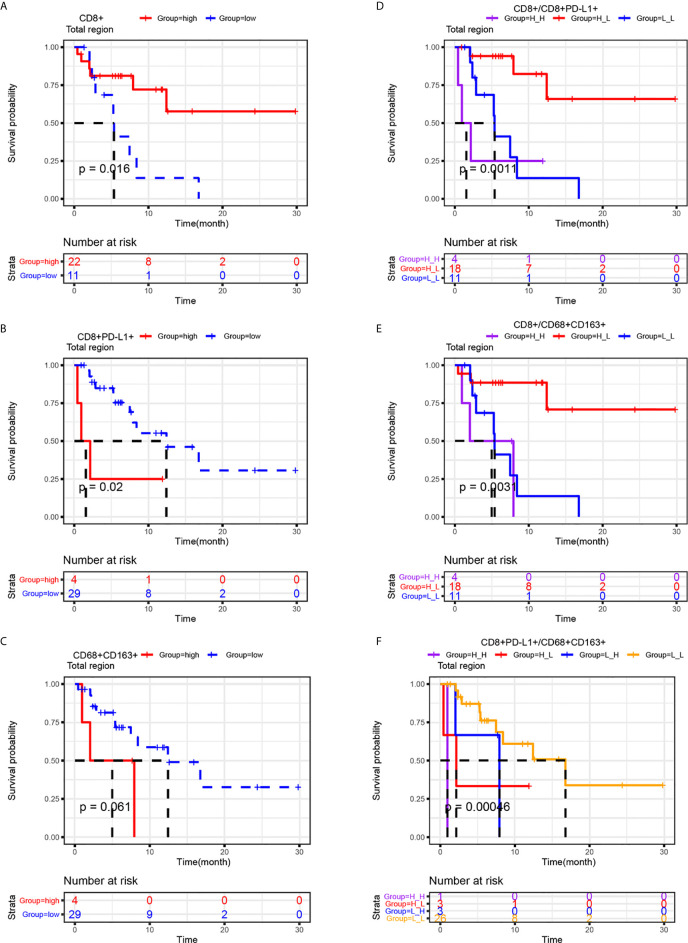
Prognostic roles of CD8+, CD8+PD-L1+, CD68+CD163+ and related joint parameters. Kaplan-Meier analysis of the progression-free survival of NSCLC patients stratified by their CD8+ **(A)**, CD8+PD-L1+ **(B)**, CD68+CD163+ **(C)** or joint parameter results **(D-F)** for the total region. The cutoff point for TAICs (high vs low) was determined using the survminer package in R software. Higher infiltration of CD8+ and lower infiltration of CD8+PD-L1+ T cells were correlated with improved progression-free survival. The results also indicated that CD8+ high/CD8+PD-L1+ low, CD8+ high/CD68+CD163+ low, and CD8+ low/CD68+CD163+ low NSCLC patients had better PFS than the other three corresponding types of patients.

Kaplan-Meier curves were generated to study whether the infiltration of CD8+ T cells, CD8+PD-L1+ T cells or CD68+CD163+ M2 macrophages or joint parameters based on these cell types in the total region, which were selected by multivariate Cox analysis, had an impact on PFS. As shown in **Figure 4**, the Kaplan-Meier curves for PFS confirmed that high infiltration of CD8+ T cells in the total region was correlated with prolonged PFS (P=0.016), and high CD8+PD-L1+ T cell infiltration in the total region was correlated with shortened PFS (P=0.02). A similar trend was observed for CD68+CD163+ M2 macrophages, although there was no statistically significant difference (P > 0.05). As CD8+ T cells, CD8+PD-L1+ T cells and CD68+CD163+ M2 macrophages in the total region can influence clinical outcomes, we stratified the patients according to joint parameters (CD8+/CD8+PD-L1+, CD8+/CD68+CD163+, and CD8+PD-L1+/CD68+CD163+). Patients with high infiltration of CD8+ T cells and low infiltration of CD8+PD-L1+ T cells in the total region had better PFS than those with any of the other three patterns (P<0.01). Analogously, patients with high infiltration of CD8+ T cells and low infiltration of CD68+CD163+ M2 macrophages in the total region had better PFS than those with any of the other three patterns (P<0.01). A similar result was observed in patients with low infiltration of CD8+ PD-L1+ T cells and low infiltration of CD68+CD163+ M2 macrophages in the total region (P<0.001). This indicates that CD8+ T cells, CD8+PD-L1+ T cells, CD8+/CD8+PD-L1+, CD8+/CD68+CD163+, and CD8+PD-L1+/CD68+CD163+ are potential biomarkers for predicting PFS in NSCLC patients receiving ICI therapy and that the CD8+/CD8+PD-L1+ and CD8+/CD68+CD163+ signatures provide better stratification of PFS than CD8+ T cells, CD8+PD-L1+ T cells, or CD68+CD163+ M2 macrophages.

### Infiltration of T Cells and M2 Macrophages Was Correlated With the Response to ICI Therapy

**Figure 5:**
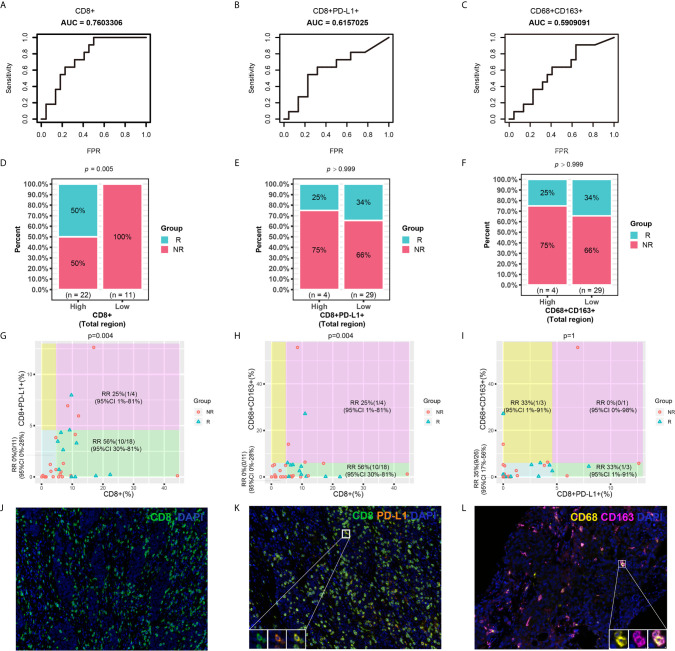
Predictive roles of CD8+, CD8+PD-L1+, CD68+CD163+ and related joint parameters. **(A–C)** ROC curves for CD8+, CD8+PD-L1+, and CD68+CD163+. **(D–F)** Chi-square tests for CD8+, CD8+PD-L1+, and CD68+CD163. **(G–I)** Scatter plot and chi-square test for joint parameters. **(J–L)** Representative mIHC images of CD8+, CD8+PD-L1+, and CD68+CD163+ cell infiltration. NSCLC patients with higher CD8+ and lower CD8+PD-L1+ and CD68+CD163+ cell infiltration were more likely to respond to ICI therapy than patients with other cell infiltration patterns. RR, response rate.

In this study, it was found that the levels of infiltrated CD8+ T cells, CD8+PD-L1+ T cells and CD68+CD163+ M2 macrophages were independent prognostic factors for PFS. However, whether they can also predict the response to ICI therapy needs further study. Receiver operating characteristic (ROC) curve analysis revealed the better predictive performance of the infiltration of CD8+ T cells (AUC=0.76) or CD8+PD-L1+ T cells (AUC=0.62) than that of CD68+CD163+ M2 macrophages (AUC=0.59) (**Figures 5A–C**). Chi-square tests were performed to study the relationships between CD8+ T cell, CD8+PD-L1+ T cell, or CD68+CD163+ M2 macrophage infiltration and the response rate of NSCLC patients who received ICI therapy. As shown in **Figures 5D–F**, the results showed that the response rate for ICI treatment in NSCLC patients with high-density infiltration of CD8+ T cells was significantly higher than that in those with low-density infiltration (P<0.01). In the total region, the response rate for ICI treatment was lower in the CD8+PD-L1+ T cell high-density infiltration subgroup than in the low-density infiltration group, but no significant difference was found (P>0.05). A similar trend was found for CD68+CD163+ M2 macrophages. A scatter plot and the chi-square test showed that the response rate in CD8+ high/CD8+PD-L1+ low subsets was 56%, which was significantly higher than that in the other two subsets (P<0.01) (**Figure 5G**). Patients with high CD8 T cell and low CD68+CD163+ M2 macrophage infiltration had a higher response rate than other patients (P<0.01) (**Figure 5H**). However, there were no significant differences identified by the CD8+PD-L1+/CD68+CD163+ parameter (**Figure 5I**). There were some examples in which a patient who responded to immunotherapy had high infiltration of CD8+ T cells and low infiltration of CD8+PD-L1+ T cells and CD68+CD163+ M2 macrophages (**Figures 5J–L**). This suggests that NSCLC patients with high infiltration of CD8+ T cells and low infiltration of CD8+PD-L1+ T cells or high infiltration of CD8+ T cells and low infiltration of CD68+CD163+ M2 macrophages were more likely to respond to ICIs than patients with other biomarker patterns. The CD8+/CD8+PD-L1+ and CD8+/CD68+CD163+ signatures provided greater stratification of the response rate than CD8+ T cells, CD8+PD-L1+ T cells, or CD68+CD163+ M2 macrophages alone.

## Discussion

This study demonstrates a relationship between TAICs in the tumor immune microenvironment and clinical outcomes in 33 NSCLC patients treated with ICIs. Our data suggest that the levels of infiltrating CD8+ T cells, CD8+PD-L1+ T cells, and CD68+CD163+ M2 macrophages in the total region were independent prognostic factors for PFS in NSCLC patients treated with ICIs. The joint parameters CD8+/CD8+PD-L1+, CD8+/CD68+CD163+, and CD8+PD-L1+/CD68+CD163+ were also potential indicators for predicting PFS in NSCLC patients receiving ICIs. In addition, the infiltration of CD8+ T cells, the combination of CD8+ and CD8+PD-L1+ T cells, and the combination of CD8+ T cells and CD68+CD163+ M2 macrophages were potential indicators for predicting the response to ICIs in NSCLC.

Our study underlines the prognostic and predictive roles of CD8+PD-L1+ T cells, CD8+ T cells, CD68+CD163+ M2 macrophages and related joint parameters in NSCLC. Our study found that NSCLC patients with high CD8+ PD-L1+ T cell infiltration had relatively poor PFS (**Figures 2** **–** **4**). Recently, some studies have investigated the relationships between the expression of PD-1 or PD-L1 on TAICs and the prognosis of ICI therapy, and high infiltration of CD8+PD-1+ T cells was found to indicate better OS or an increased response rate (13, 25, 28, 29). Since PD-L1 expressed on tumor cells and macrophages is a negative regulator of T cell responses, blocking the PD-1/PD-L1 axis can improve immune responses against tumors (24); furthermore, it makes sense that in a 62-person cohort of NSCLC patients treated with ICIs, high levels of CD68+PD-L1+ immune cells were associated with prolonged OS (25). However, few studies have reported the relationship between the expression of PD-L1 on T cells and immunotherapy. It has been reported previously that PD-L1^high^ CD8+ T cells express more CD107a and IFN-γ than PD-L1^low^CD8+ T cells, which indicates that PD-L1^high^ CD8+ T cells are functional effector cells (26). However, the ligation of PD-L1 in T cells can induce IL-10 expression and T cell apoptosis (30). A recent pancreatic cancer study showed that PD-L1+ T cells exerted tumor-promoting tolerance in 3 ways: PD-L1+ T cells could prevent activation, reduce Th1 polarization and promote Th17 differentiation (31). Through the PD-L1–PD-1 axis, PD-L1+ T cells can suppress effector T cells even without endogenous PD-L1 (31). The engagement of PD-L1+ T cells and PD-1+ macrophages can induce M2 macrophages, which have negative effects on adaptive antitumor immunity (31). This may explain why NSCLC patients with high levels of CD8+PD-L1+ T cells have relatively poor PFS. These results indicate that CD8+ PD-L1+ T cells are a risk factor for ICI therapy in NSCLC.

The present work identified the value of CD8+ T cells in predicting PFS and treatment response (**Figures 2** **–** **5**). Multiple studies have shown that high infiltration of CD8+ T cells correlates with improved survival in patients treated with ICIs (18, 32, 33). It was reported that a high density of CD8+ cells was associated with a higher median OS time in 163 NSCLC patients who received durvalumab (P<0.01) (32). Some studies have also found that high infiltration of CD8+ T cells is correlated with a relatively good response in patients treated with ICIs (12, 19–21). A previous study found that the level of CD8+ T cells was significantly higher in patients achieving CR/PR than in those with SD/PD in melanoma (P < 0.0001) (32). Our experimental results confirmed that CD8+ T cells are a good biomarker for predicting the response and survival of patients treated with ICIs.

Our data revealed that NSCLC patients with high CD68+ CD163+ macrophage infiltration had relatively poor PFS (**Figure 3**). Macrophages in the tumor microenvironment are defined as TAMs, which can produce growth factors, cytokines, and other molecules to regulate metastasis (22). The M1 and M2 states are two main phenotypes of macrophages. It has been reported that CD68+CD163- cells and CD68+CD163+ cells are considered M1-like macrophages and M2-like macrophages, respectively (23). Low infiltration of M1-like macrophages and high infiltration of M2-like macrophages are strongly associated with poor disease-free survival (23). M2-like macrophages have repair- and growth-inducing properties that can promote tumor progression, angiogenesis and metastasis (23, 34–36). This may explain the reason why high levels of CD68+CD163+ M2 macrophages were associated with poorer PFS than low levels of CD68+CD163+ M2 macrophages in this cohort.

Because of the prognostic and predictive roles of CD8+PD-L1+ T cells, CD8+ T cells, and CD68+CD163+ M2 macrophages, it was unsurprising to find that NSCLC patients with a CD8+ high/CD8+PD-L1+ low, CD8+ high/CD68+CD163+ low or CD8+PD-L1+ high/CD8+PD-L1+ low signature had better PFS than patients with the other corresponding signatures (**Figure 4**). We also found that the response rate in NSCLC patients with a CD8+ high/CD8+PD-L1+ low or CD8+ high/CD68+CD163+ low signature was significantly higher than that in patients with the other corresponding signatures (**Figure 5**). Overall, for PFS, the joint parameters CD8+/CD8+PD-L1+, CD8+/CD68+CD163+ and CD8+PD-L1+/CD8+PD-L1+ demonstrated stronger stratification than the single biomarkers. Regarding the response rate, the joint parameters CD8+/CD8+PD-L1+ and CD8+/CD68+CD163+ also demonstrated stronger stratification than the single biomarkers. Our findings suggest that joint evaluation of multiple biomarkers has certain significance in studying the immune status of tumors and guiding ICI treatment in NSCLC patients.

There were several limitations of this study. First, the analysis of this study was based on 33 NSCLC patients. The predictive values of CD8+PD-L1+ T cells, CD8+ T cells, CD68+CD163+ M2 macrophages and related joint parameters need to be validated in larger studies. Second, the molecular characteristics of these biomarkers, especially CD8+PD-L1+ T cells, need further study.

In summary, our retrospective study revealed the prognostic and predictive value of CD8+PD-L1+ T cells, CD8+ T cells, and CD68+CD163+ M2 macrophages in NSCLC patients who received ICIs. These biomarkers are likely to predict response and survival. Interestingly, our results indicate that evaluation of joint parameters composed of these biomarkers has certain significance for guiding ICI treatment in NSCLC. Our results warrant validation and further study.

## Data Availability Statement

The raw data supporting the conclusions of this article will be made available by the authors, without undue reservation.

## Ethics Statement

The studies involving human participants were reviewed and approved by the Ethics Committee of Guangdong Hospital of Traditional Chinese Medicine. The patients/participants provided their written informed consent to participate in this study. Written informed consent was obtained from the individual(s) for the publication of any potentially identifiable images or data included in this article.

## Author Contributions

Conceptualization: LL, GL, YL, WG, and LY. Methodology: LL, GL, and YL. Software: LL, GL, and YL. Formal analysis: LL, GL, and YL. Investigation: LL, GL, YL, LG, and XZ. Resources: LL and GL. Data curation: LL, WG, GL, YL, and XZ. Writing - original draft preparation: LL, GL, and YL. Writing - review and editing: LL, GL, YL, LG, XZ, HZ, LY, and WG. Visualization: LL, GL, and YL. Supervision: LL, GL, and YL. All authors contributed to the article and approved the submitted version.

## Conflict of Interest

YL, LG, XZ and HZ were employed by Genecast Biotechnology Co., Ltd.

The authors declare that the research was conducted in the absence of any commercial or financial relationships that could be constructed as a potential conflict of interest.

## References

[1] Gettinger SN Horn L Gandhi L Spigel DR Antonia SJ Rizvi NA. Overall Survival and Long-Term Safety of Nivolumab (Anti-Programmed Death 1 Antibody, Bms-936558, ONO-4538) in Patients With Previously Treated Advanced Non-Small-Cell Lung Cancer. J Clin Oncol (2015) 33(18):2004–12. 10.1200/jco.2014.58.3708 PMC4672027 25897158

[2] Garon EB Rizvi NA Hui R Leighl N Balmanoukian AS Eder JP. Pembrolizumab for the Treatment of Non-Small-Cell Lung Cancer. N Engl J Med (2015) 372(21):2018–28. 10.1056/NEJMoa1501824 25891174

[3] Reck M Rodríguez-Abreu D Robinson AG Hui R Csőszi T Fülöp A. Pembrolizumab Versus Chemotherapy for PD-L1-Positive Non-Small-Cell Lung Cancer. N Engl J Med (2016) 375(19):1823–33. 10.1056/NEJMoa1606774 27718847

[4] Rittmeyer A Barlesi F Waterkamp D Park K Ciardiello F von Pawel J. Atezolizumab Versus Docetaxel in Patients With Previously Treated Non-Small-Cell Lung Cancer (OAK): A Phase 3, Open-Label, Multicentre Randomised Controlled Trial. Lancet (2017) 389(10066):255–65. 10.1016/s0140-6736(16)32517-x PMC6886121 27979383

[5] Antonia SJ Villegas A Daniel D Vicente D Murakami S Hui R. Durvalumab After Chemoradiotherapy in Stage III Non-Small-Cell Lung Cancer. N Engl J Med (2017) 377(20):1919–29. 10.1056/NEJMoa1709937 28885881

[6] Herbst RS Soria JC Kowanetz M Fine GD Hamid O Gordon MS. Predictive Correlates of Response to the Anti-PD-L1 Antibody MPDL3280A in Cancer Patients. Nature (2014) 515(7528):563–7. 10.1038/nature14011 PMC4836193 25428504

[7] National Comprehensive Cancer Network. (NCCN) Clinical Practice Guidelines in Oncology, in: Non-Small Cell Lung Cancer, Version 5 (2020). Available at: https://www.nccn.org/store/login/login.aspx?ReturnURL=https://www.nccn.org/professionals/physician_gls/pdf/nscl.pdf (Accessed 27 May 2020).

[8] Topalian SL Hodi FS Brahmer JR Gettinger SN Smith DC McDermott DF. Safety, Activity, and Immune Correlates of Anti-PD-1 Antibody in Cancer. N Engl J Med (2012) 366(26):2443–54. 10.1056/NEJMoa1200690 PMC3544539 22658127

[9] Mok TSK Wu YL Kudaba I Kowalski DM Cho BC Turna HZ. Pembrolizumab Versus Chemotherapy for Previously Untreated, PD-L1-Expressing, Locally Advanced or Metastatic Non-Small-Cell Lung Cancer (KEYNOTE-042): A Randomised, Open-Label, Controlled, Phase 3 Trial. Lancet (2019) 393(10183):1819–30. 10.1016/s0140-6736(18)32409-7 30955977

[10] Hellmann MD Ciuleanu TE Pluzanski A Lee JS Otterson GA Audigier-Valette C. Nivolumab Plus Ipilimumab in Lung Cancer With a High Tumor Mutational Burden. N Engl J Med (2018) 378(22):2093–104. 10.1056/NEJMoa1801946 PMC7193684 29658845

[11] Carbone DP Reck M Paz-Ares L Creelan B Horn L Steins M. First-Line Nivolumab in Stage IV or Recurrent Non-Small-Cell Lung Cancer. N Engl J Med (2017) 376(25):2415–26. 10.1056/NEJMoa1613493 PMC6487310 28636851

[12] Haratani K Hayashi H Tanaka T Kaneda H Togashi Y Sakai K. Tumor Immune Microenvironment and Nivolumab Efficacy in EGFR Mutation-Positive Non-Small-Cell Lung Cancer Based on T790M Status After Disease Progression During EGFR-TKI Treatment. Ann Oncol (2017) 28(7):1532–9. 10.1093/annonc/mdx183 28407039

[13] Thommen DS Koelzer VH Herzig P Roller A Trefny M. A Transcriptionally and Functionally Distinct PD-1(+) Cd8(+) T Cell Pool With Predictive Potential in non-Small-Cell Lung Cancer Treated With PD-1 Blockade. Nat Med (2018) 24(7):994–1004. 10.1038/s41591-018-0057-z 29892065 PMC6110381

[14] Garassino M Rodriguez-Abreu D Gadgeel S Esteban E Felip E Speranza G. Oa04.06 Evaluation of TMB in KEYNOTE-189: Pembrolizumab Plus Chemotherapy vs Placebo Plus Chemotherapy for Nonsquamous Nsclc. J Thorac Oncol (2019) 14:S216–7. 10.1016/j.jtho.2019.08.427

[15] Paz-Ares L Langer C Novello S Halmos B Cheng Y Gadgeel S. LBA80Pembrolizumab (Pembro) Plus Platinum-Based Chemotherapy (Chemo) for Metastatic NSCLC: Tissue TMB (tTMB) and Outcomes in KEYNOTE-021, 189, and 407. Ann Oncol (2019) 30:v917–8. 10.1093/annonc/mdz394.078

[16] Fridman WH Pagès F Sautès-Fridman C Galon J. The Immune Contexture in Human Tumours: Impact on Clinical Outcome. Nat Rev Cancer (2012) 12(4):298–306. 10.1038/nrc3245 22419253

[17] Bruni D Angell HK. The Immune Contexture and Immunoscore in Cancer Prognosis and Therapeutic Efficacy. Nat Rev Cancer (2020) 20(11):662–80. 10.1038/s41568-020-0285-7 32753728

[18] Althammer S Tan TH Spitzmüller A Rognoni L Wiestler T Herz T. Automated Image Analysis of NSCLC Biopsies to Predict Response to Anti-PD-L1 Therapy. J Immunother Cancer (2019) 7(1):121. 10.1186/s40425-019-0589-x 31060602 PMC6501300

[19] Schmid P Salgado R Park YH Muñoz-Couselo E Kim SB Sohn J. Pembrolizumab Plus Chemotherapy as Neoadjuvant Treatment of High-Risk, Early-Stage Triple-Negative Breast Cancer: Results From the Phase 1B Open-Label, Multicohort KEYNOTE-173 Study. Ann Oncol (2020) 31(5):569–81. 10.1016/j.annonc.2020.01.072 32278621

[20] Chen Y Wang Y Luo H Meng X Zhu W Wang D. The Frequency and Inter-Relationship of PD-L1 Expression and Tumour Mutational Burden Across Multiple Types of Advanced Solid Tumours in China. Exp Hematol Oncol (2020) 9:17. 10.1186/s40164-020-00173-3 32775040 PMC7397649

[21] Ji S Chen H Yang K Zhang G Mao B Hu Y. Peripheral Cytokine Levels as Predictive Biomarkers of Benefit From Immune Checkpoint Inhibitors in Cancer Therapy. Biomed Pharmacother (2020) 129:110457. 10.1016/j.biopha.2020.110457 32887027

[22] Salmaninejad A Valilou SF Soltani A Ahmadi S Abarghan YJ Rosengren RJ. Tumor-Associated Macrophages: Role in Cancer Development and Therapeutic Implications. Cell Oncol (Dordr) (2019) 42(5):591–608. 10.1007/s13402-019-00453-z 31144271 PMC12994359

[23] Schnellhardt S Erber R. Accelerated Partial Breast Irradiation: Macrophage Polarisation Shift Classification Identifies High-Risk Tumours in Early Hormone Receptor-Positive Breast Cancer. Cancers (2020) 12(2):446. 10.3390/cancers12020446 PMC7072550 32075091

[24] Keir ME Butte MJ Freeman GJ Sharpe AH. PD-1 and Its Ligands in Tolerance and Immunity. Annu Rev Immunol (2008) 26:677–704. 10.1146/annurev.immunol.26.021607.090331 18173375 PMC10637733

[25] Liu Y Zugazagoitia J Ahmed FS Henick BS Gettinger SN Herbst RS. Immune Cell Pd-L1 Colocalizes With Macrophages and Is Associated With Outcome in PD-1 Pathway Blockade Therapy. Clin Cancer Res (2020) 26(4):970–7. 10.1158/1078-0432.ccr-19-1040 PMC7024671 31615933

[26] Liu X Wu X Cao S Harrington SM Yin P Mansfield AS. B7-H1 Antibodies Lose Antitumor Activity Due to Activation of P38 MAPK That Leads to Apoptosis of Tumor-Reactive CD8(+) T Cells. Sci Rep (2016) 6:36722. 10.1038/srep36722 27824138 PMC5099859

[27] Gorris MAJ Halilovic A Rabold K. Eight-Color Multiplex Immunohistochemistry for Simultaneous Detection of Multiple Immune Checkpoint Molecules Within the Tumor Microenvironment. J Immunol (2018) 200(1):347–54. 10.4049/jimmunol.1701262 29141863

[28] Chalabi M Fanchi LF Dijkstra KK Van den Berg JG Aalbers AG Sikorska K. Neoadjuvant Immunotherapy Leads to Pathological Responses in MMR-proficient and MMR-deficient Early-Stage Colon Cancers. Nat Med (2020) 26: (4):566–76. 10.1038/s41591-020-0805-8 32251400

[29] Terranova-Barberio M Pawlowska N Dhawan M Moasser M. Exhausted T Cell Signature Predicts Immunotherapy Response in ER-Positive Breast Cancer. Nat Comm (2020) 11: (1):3584. 10.1038/s41467-020-17414-y PMC7367885 32681091

[30] Dong H Strome SE Matteson EL Moder KG Flies DB Zhu G. Costimulating Aberrant T Cell Responses by B7-H1 Autoantibodies in Rheumatoid Arthritis. J Clin Invest (2003) 111(3):363–70. 10.1172/jci16015 PMC151851 12569162

[31] Diskin B Adam S Cassini MF Sanchez G Liria M Aykut B. Pd-L1 Engagement on T Cells Promotes Self-Tolerance and Suppression of Neighboring Macrophages and Effector T Cells in Cancer. Nat Immunol (2020) 21(4):442–54. 10.1038/s41590-020-0620-x 32152508

[32] Wong PF Wei W Smithy JW. Multiplex Quantitative Analysis of Tumor-Infiltrating Lymphocytes and Immunotherapy Outcome in Metastatic Melanoma. Clin Cancer Res (2019) 25: (8):2442–9. 10.1158/1078-0432.ccr-18-2652 PMC6467753 30617133

[33] Emens LA Cruz C Eder JP Braiteh F Chung C Tolaney SM. Long-Term Clinical Outcomes and Biomarker Analyses of Atezolizumab Therapy for Patients With Metastatic Triple-Negative Breast Cancer: A Phase 1 Study. JAMA Oncol (2019) 5(1):74–82. 10.1001/jamaoncol.2018.4224 30242306 PMC6439773

[34] Mantovani A Sozzani S Locati M Allavena P Sica A. Macrophage Polarization: Tumor-Associated Macrophages as a Paradigm for Polarized M2 Mononuclear Phagocytes. Trends Immunol (2002) 23(11):549–55. 10.1016/s1471-4906(02)02302-5 12401408

[35] Grivennikov SI Wang K Mucida D Stewart CA Schnabl B Jauch D. Adenoma-Linked Barrier Defects and Microbial Products Drive IL-23/IL-17-Mediated Tumour Growth. Nature (2012) 491(7423):254–8. 10.1038/nature11465 PMC3601659 23034650

[36] Kong L Zhou Y Bu H Lv T Shi Y Yang J. Deletion of Interleukin-6 in Monocytes/Macrophages Suppresses the Initiation of Hepatocellular Carcinoma in Mice. J Exp Clin Cancer Res (2016) 35(1):131. 10.1186/s13046-016-0412-1 27589954 PMC5009700

