# Supplementary material for: Low Infiltration of CD8+ PD-L1+ T Cells and M2 Macrophages Predicts Improved Clinical Outcomes After Immune Checkpoint Inhibitor Therapy in Non-Small Cell Lung Carcinoma
Source: Front Oncol. 2021 Jun 4;11:658690. doi: 10.3389/fonc.2021.658690 (PMC8213070; doi:10.3389/fonc.2021.658690)
Supplement: Supplementary file 1 [file DataSheet_1.docx]

**Supplementary Material**

**
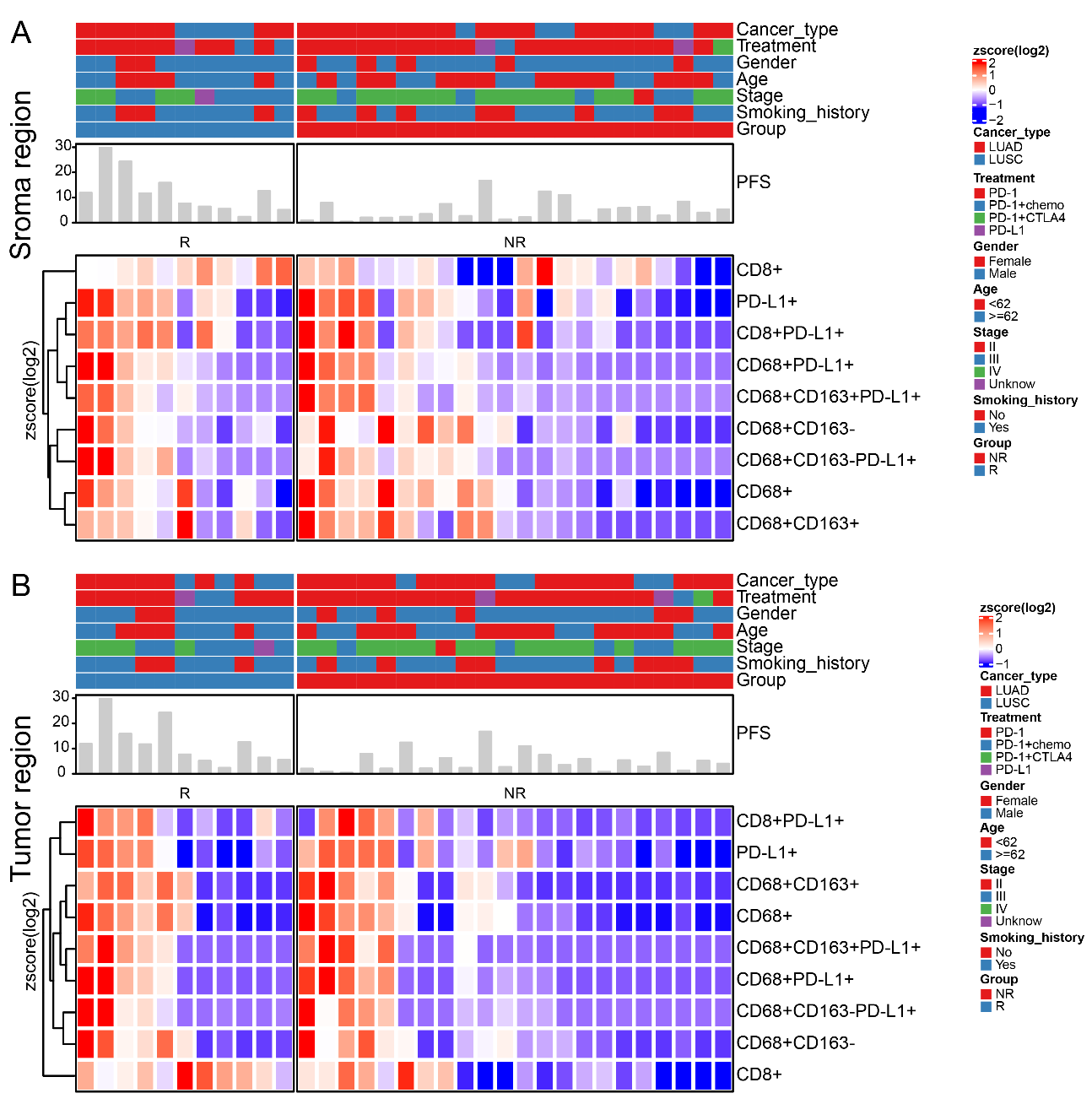
**

**Figure S1. Immune landscape of NSCLC patients treated with immune checkpoint inhibitor (ICI) therapy.** Immune cell infiltration was detected by using the multiplex immunohistochemistry (mIHC) platform. The percentages of differentially expressed cells were log-transferred and z-scored standardized. Heatmaps of immune cell infiltration in the stroma (A) and tumor region (B) were plotted and clustered. R: response, NR: non-response.

**
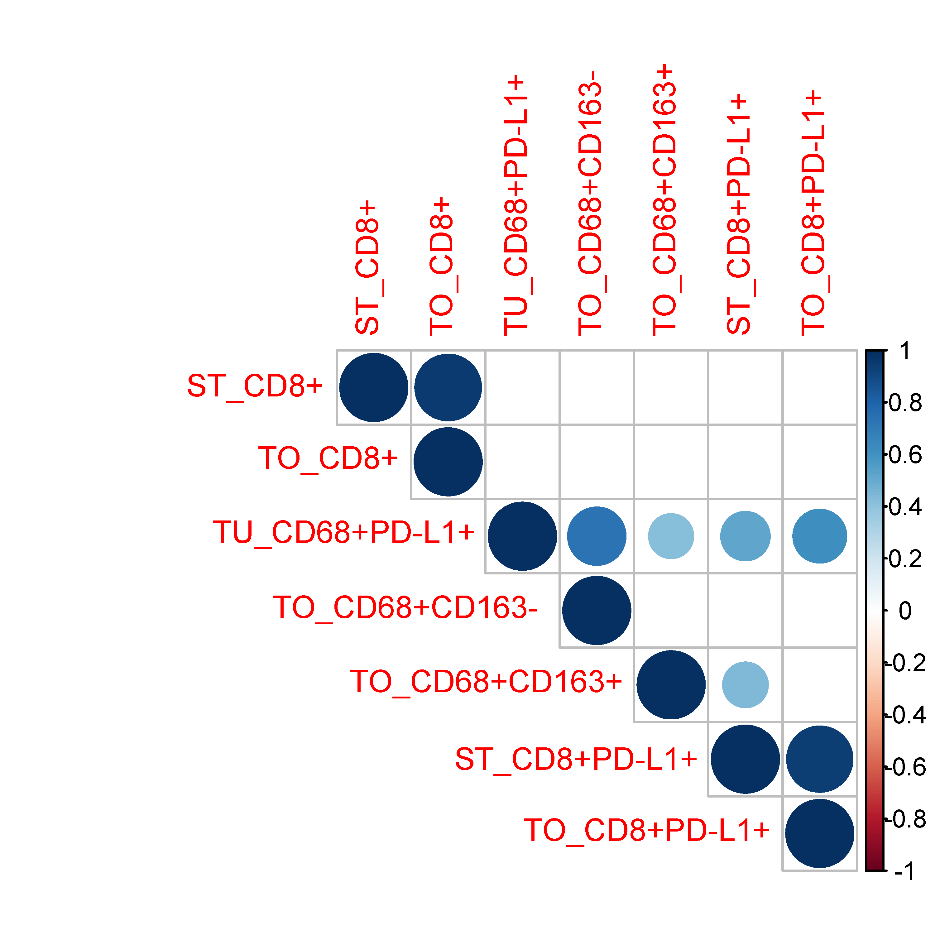
**

**Figure S2. Correlation analysis of immune cell subsets.** The correlation coefficient of CD8+ between total region and stroma region, and the correlation coefficient of CD8+PD-L1+ between total region and stroma region were greater than 0.9. ST: stroma region, TU: tumor region, TO: total region.
